# Pilomatricoma in the infraorbital region

**DOI:** 10.1002/ccr3.9322

**Published:** 2024-08-12

**Authors:** Dilasha Dhungel, Varun Rastogi, Sandhya Chaurasia, Nisha Maddheshiya

**Affiliations:** ^1^ Department of Oral and Maxillofacial Pathology Universal College of Medical Sciences Bhairahawa Nepal; ^2^ Banaras Hindu University Institute of Medical Sciences Varanasi India

**Keywords:** basaloid cells, cytokeratin, hair follicles, infraorbital region, Pilomatricoma, shadow cells/ghost cells, skin tumor

## Abstract

**Key Clinical Message:**

Pilomatricoma, a rare benign skin tumor arising from hair follicle matrix cells, warrants consideration in the evaluation of subcutaneous nodules or masses, especially when presenting as painless and firm lesions. Accurate diagnosis hinges on histopathological examination, underscoring the significance of clinician vigilance and prompt intervention.

**Abstract:**

Pilomatricoma, also referred to as *Pilomatrixomas or Calcifying epithelioma of Malherbe*, is a rare benign skin tumor derived from hair follicle matrix cells, presents a diagnostic challenge due to its diverse clinical manifestations. Females are more commonly affected by Pilomatricoma. This condition typically manifests as a painless, firm, and slowly progressive lesion. Histopathological analysis shows characteristic findings, such as basaloid cells at the periphery and shadow cells centrally. Immunohistochemical studies assess the expression of cytokeratin's associated with hair matrix differentiation. Complete surgical excision remains the cornerstone of treatment, ensuring favorable outcomes and minimizing the risk of recurrence. Awareness of this entity among clinicians is essential for timely recognition and appropriate intervention. In this specific case‐study, we present a case of Pilomatricoma situated in the lower left orbital region of a 32‐year‐old male individual who had been noticing swelling in that vicinity over the preceding 7 months.

## 1 INTRODUCTION

Pilomatricoma is a relatively rare benign neoplasm of the hair follicle matrix cells.^1^, ^2^, ^3^ In 1880, Malherbe and Chenantais were the first to describe Pilomatricoma.^4^ Pilomatricoma occurs in about 1% of all benign skin lesions.^5^ It primarily manifests in children and adolescents, with a slight preference for females.^1^, ^3^ The head and neck area (40%–70% cases) is the most frequent site for Pilomatricoma, followed by the upper extremities, trunk, and lower extremities.^2^, ^6^ It presents as asymptomatic, firm, solitary, slow‐growing, painless nodule along with superficial ulceration, or bluish discoloration.^1^ Typically, Pilomatricoma present as singular lesions, although multiple lesions can occur in association with conditions such as Steinert myotonic dystrophy, familial adenomatosis, Rubinstein‐Taybi syndrome, Gardner's syndrome, Turner syndrome, and sarcoidosis.^6^, ^7^ The report provides a comprehensive overview of a case concerning a 32‐year‐old male patient who was diagnosed with a Pilomatricoma situated in the left infraorbital region.

## 2 CASE HISTORY/EXAMINATION

**FIGURE 1:**
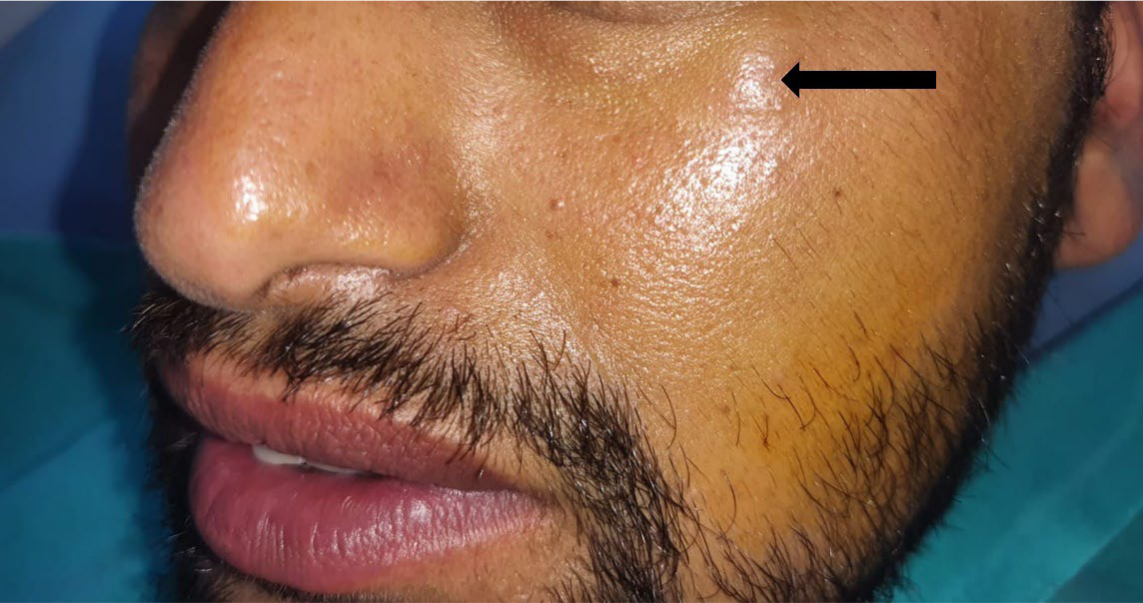
Clinical presentation.

A 32‐year‐old male visited the Department of Oral Medicine and Radiology, reporting swelling in the left infraorbital region persisting for 7 months. Upon examination, a small, soft, mobile mass measuring approximately 1 cm × 1 cm was observed in the left infraorbital rim area. The mass was non‐tender upon palpation (Figure 1).

## 3 METHODS

**FIGURE 2:**
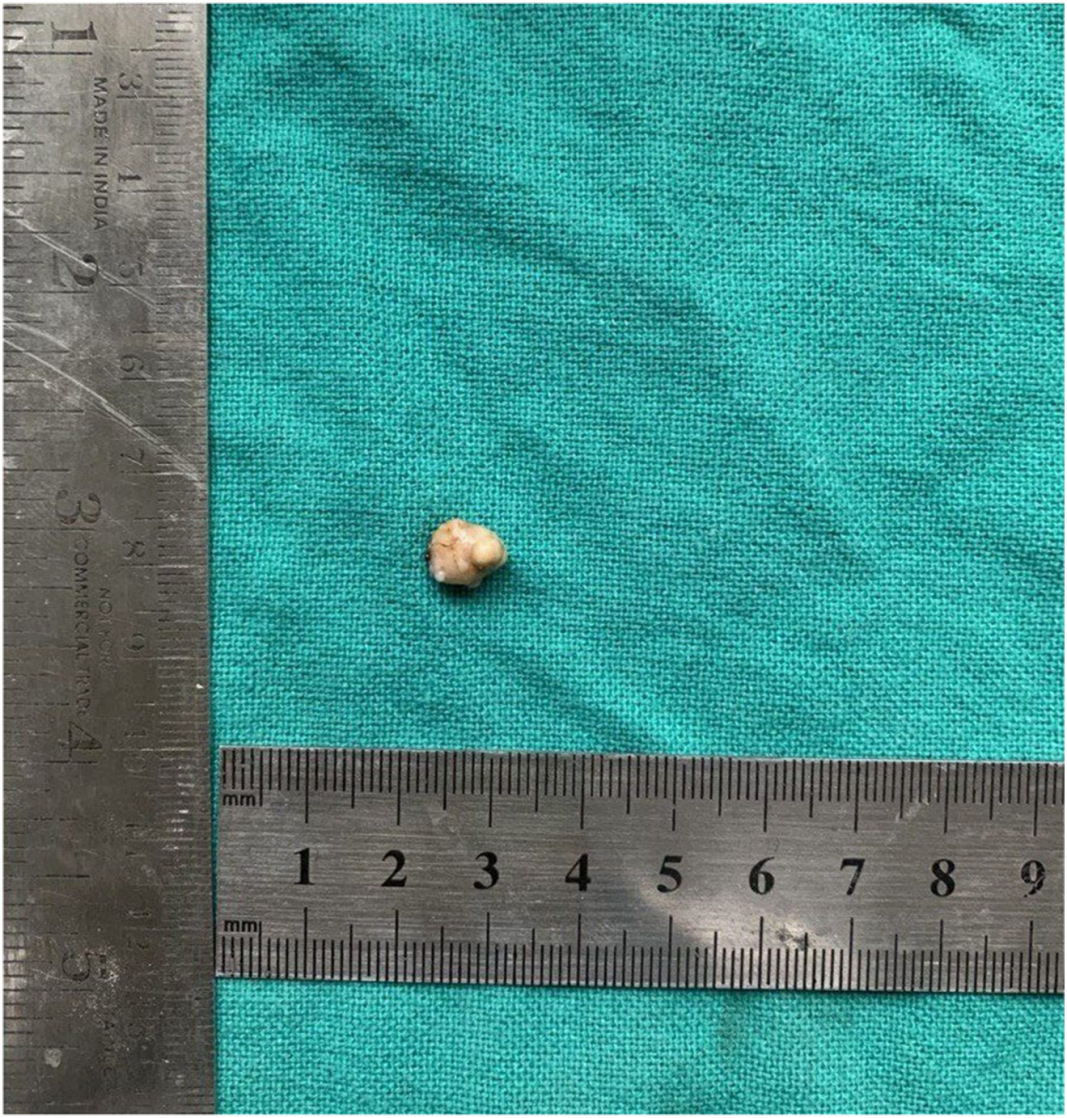
Macroscopic findings.

**FIGURE 3:**
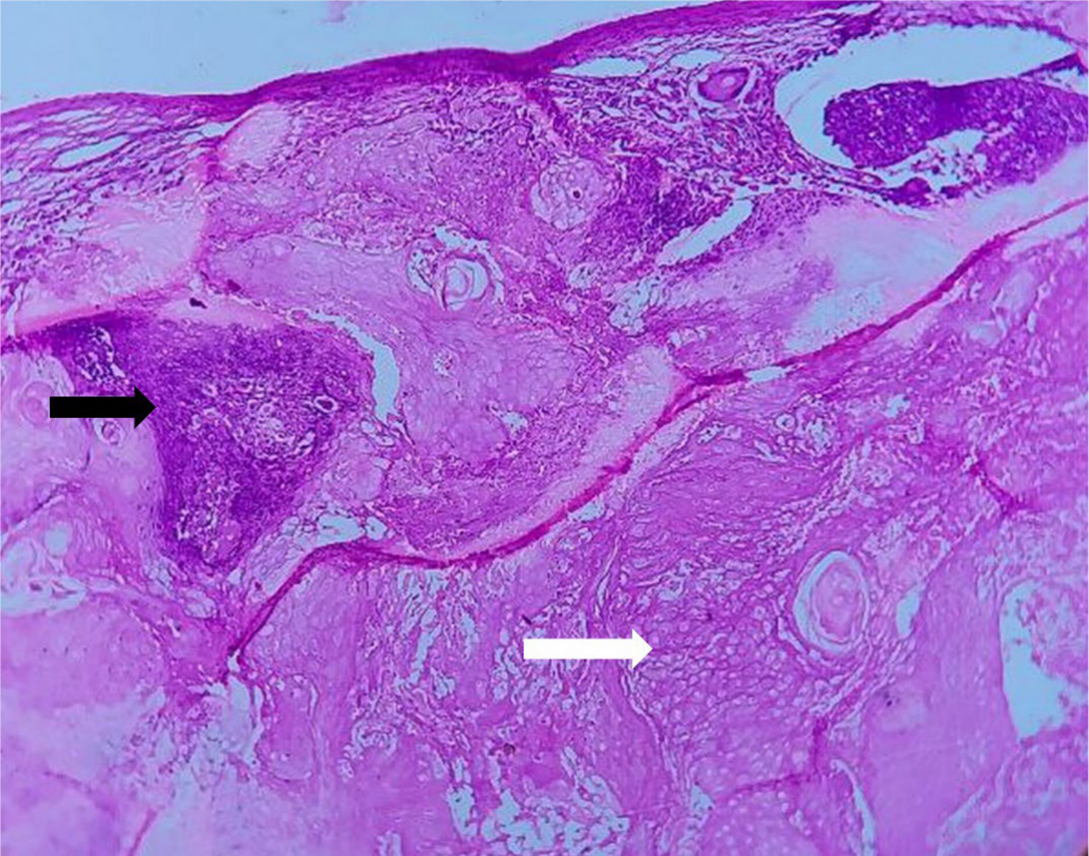
Scanner view showing islands of basaloid cells (black arrow) and ghost cells (white arrow).

**FIGURE 4:**
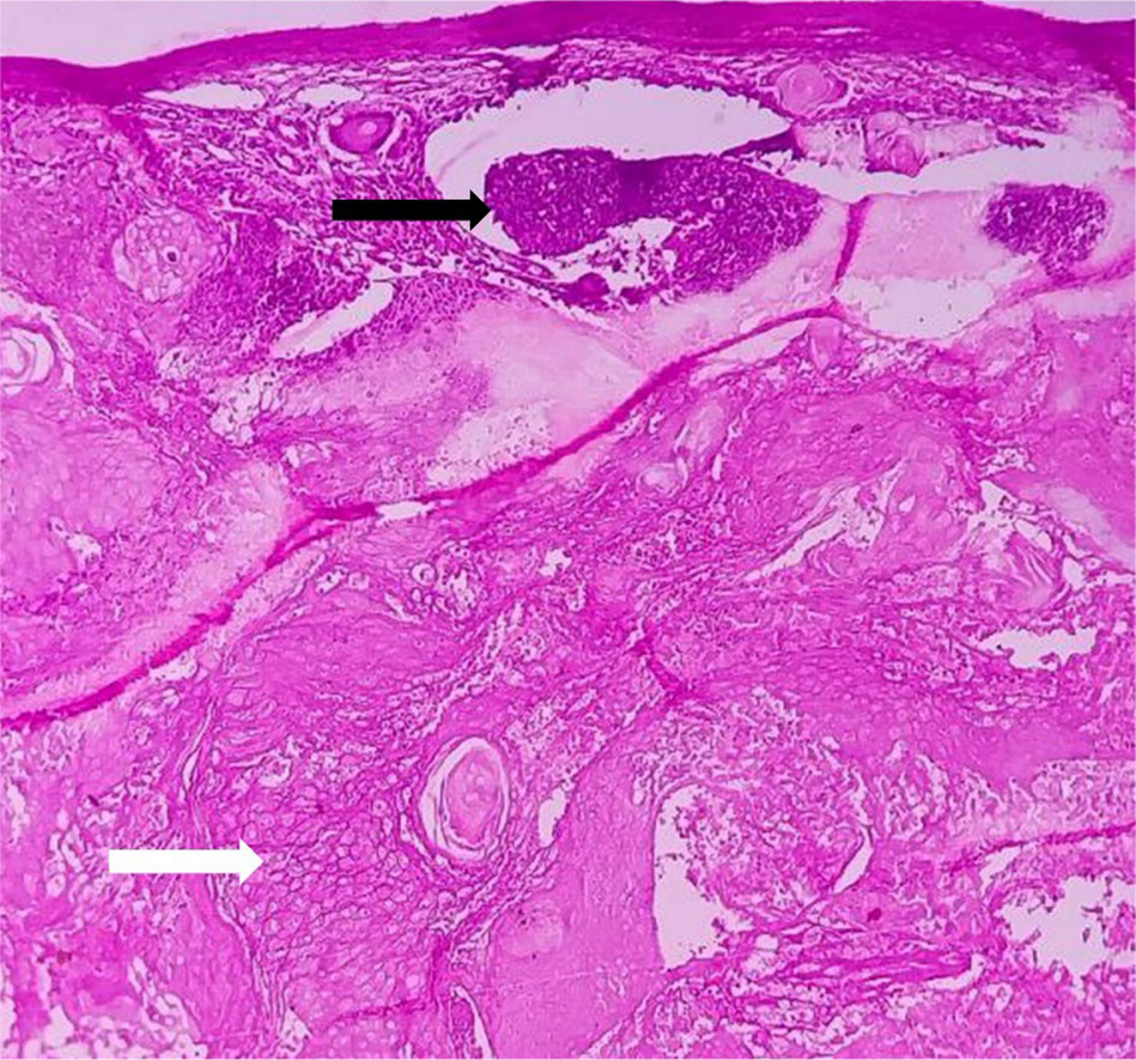
Islands of basaloid cells (black arrow) and ghost cells (white arrow) in 10x magnification.

**FIGURE 5:**
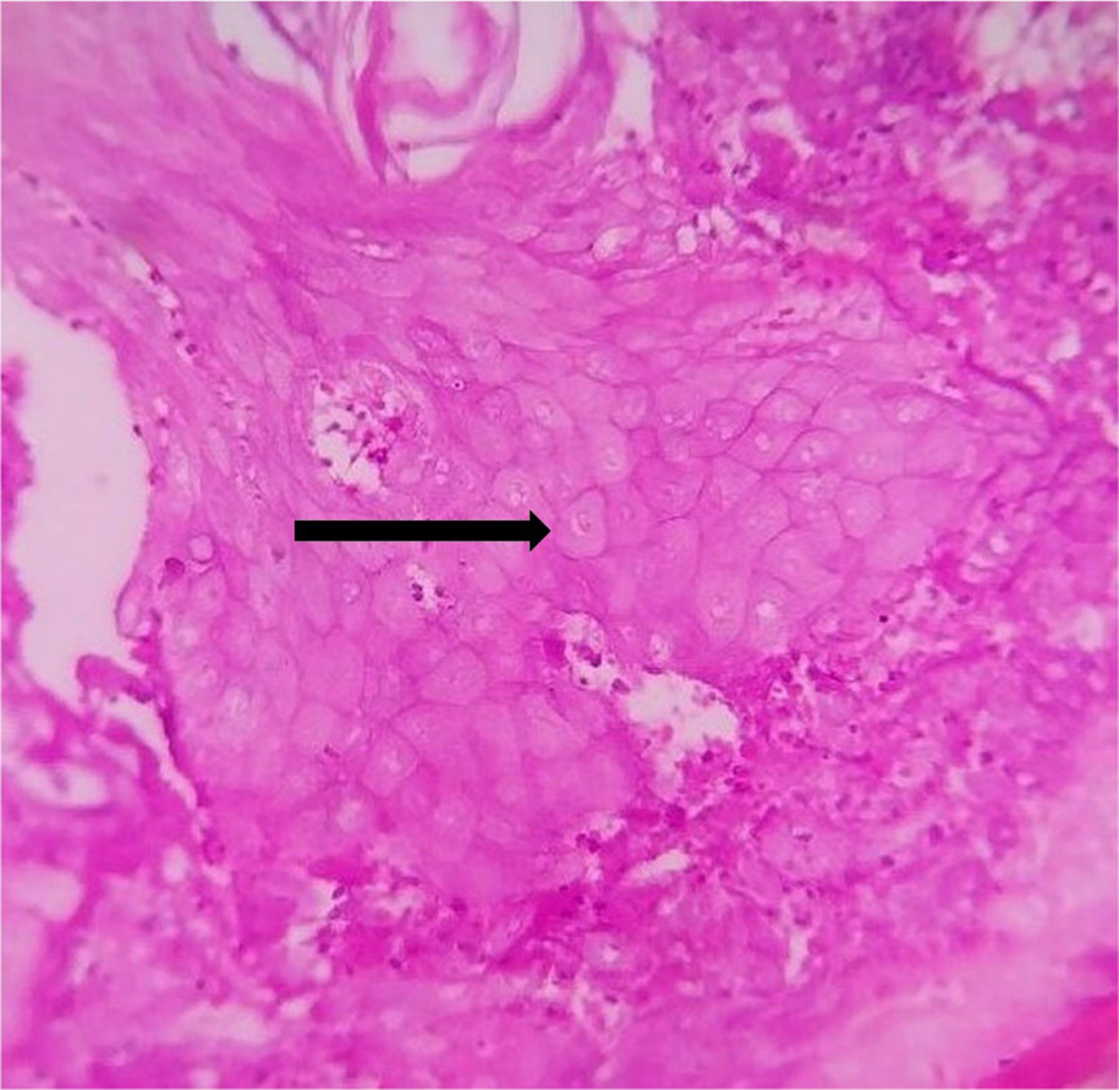
Ghost cells in 40x magnification (black arrow).

The differential diagnosis was dermoid and sebaceous cyst. Following the examination, surgical excision was performed under general anesthesia. The biopsy tissue extracted during the procedure was preserved in a 10% formalin solution and forwarded for additional histopathological analysis. Macroscopic examination revealed a single piece of tissue, exhibiting a grayish‐white to light brown hue. It displayed a firm consistency and measured approximately 0.9 cm × 0.8 cm (Figure 2). Histopathological examination by routine hematoxylin and eosin (H and E) staining shows a well circumscribed, lobulated dermal adnexal neoplasm. Upon lower magnification examination, clusters of basaloid cells with round to oval hyperchromatic nuclei and increased mitotic activity were observed (Figures 3 and 4). These cells also exhibit abrupt keratinization along with structureless eosinophilic cells lacking nuclei called as ghost cells or shadow cells (Figure 5). The surrounding stroma shows foreign body giant cell reaction, chronic inflammatory cells and moderate vascularity. After correlating the clinical presentation with the histopathological findings, the diagnosis of Pilomatricoma located in the infraorbital region was confirmed.

## 4 CONCLUSION AND RESULTS

Following complete surgical excision, the patient was monitored closely during follow‐up visits. At the 6‐month postoperative follow‐up, the patient remained free of any signs of disease recurrence.

## 5 DISCUSSION

**FIGURE 6:**
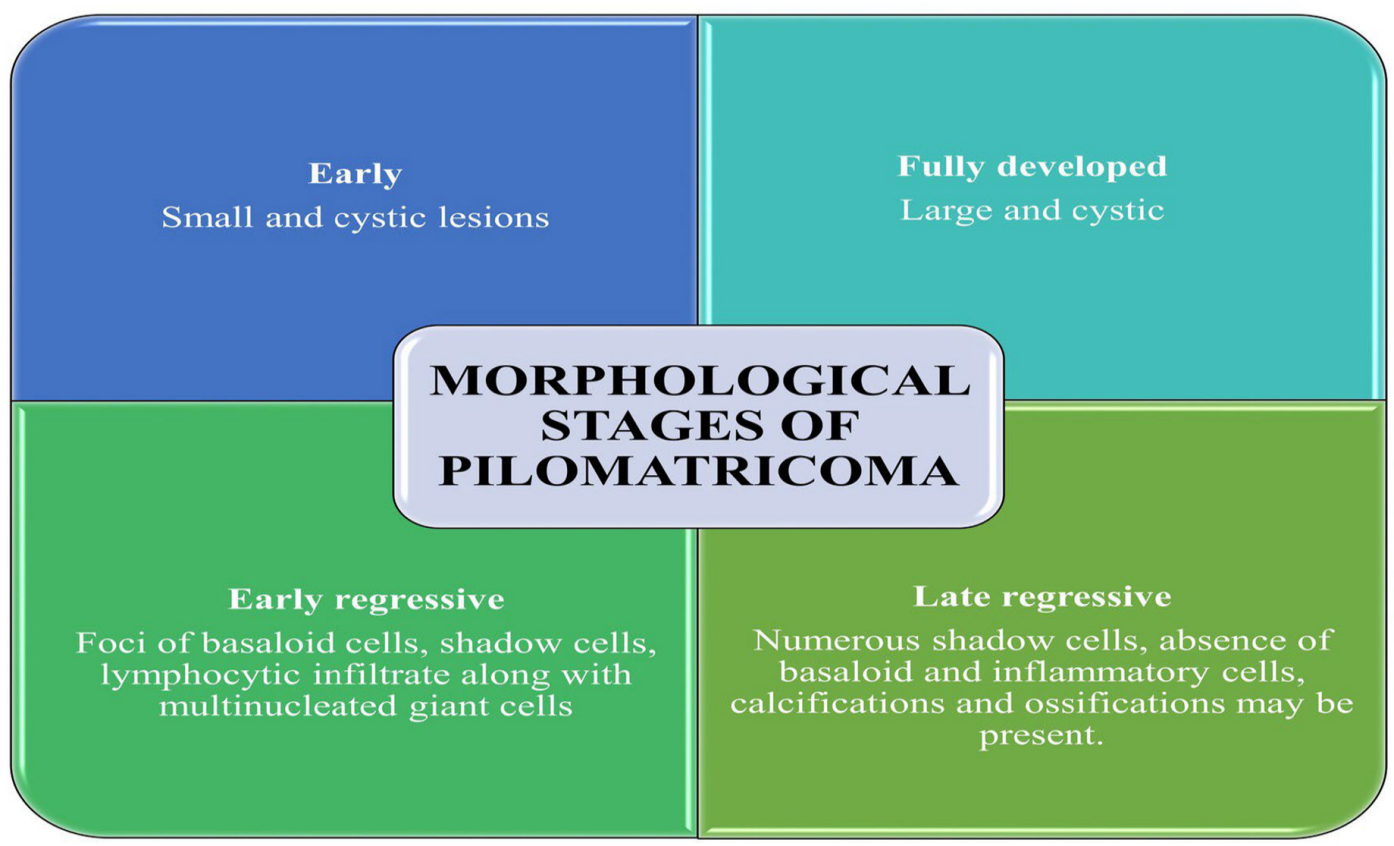
Morphological stages of pilomatricoma.

Pilomatricoma is a benign neoplasm derived from hair matrix cells associated with β‐catenin gene CTNNB1 mutation.^3^, ^8^ β‐catenin serves as a downstream mediator in the Wnt signaling pathway, regulating various cellular processes including cell differentiation within the hair follicle, cell adhesion, and cell proliferation. Mutations occurring in exon 3 of the CTNNB1 gene induce an upsurge in β‐catenin levels, consequently contributing to the formation of neoplasms characterized by hair matrix differentiation. Approximately, 26%–100% of pilomatrixomas are associated with β‐catenin gene mutations.^4^ Figure 6 outlines the four identifiable morphological phases of Pilomatricoma: early, fully developed, early regressive, and late regressive.^6^

It has bimodal age distribution, predominantly seen in the first and second decades of life and another peak in the 55–65‐year age group of patients.^4^, ^7^ During examination, the Pilomatricoma exhibits distinctive signs such as the “tent sign” when the skin is stretched over the tumor, and the “teeter‐totter sign” where pressure on one side of the lesion causes protrusion on the opposite side, resembling a seesaw motion. These signs are pathognomonic, providing crucial diagnostic indicators for pilomatricoma.^6^ The clinical presentation of Pilomatricoma may be confused with several other conditions, leading to a broad range of differential diagnoses. These include epidermoid cysts, dermoid cysts, chalazion, capillary hemangiomas, sebaceous adenoma, juvenile Xanthogranuloma, and rhabdomyosarcoma (Table 1).^8^, ^9^

**TABLE 1:** Clinical differential diagnosis.

Condition	Histopathological features
Epidermoid cyst	Keratin‐filled cyst lined by stratified squamous epithelium.
Dermoid cyst	Cyst containing skin adnexal structures like hair follicles and sebaceous glands.
Chalazion	Inflammatory reaction to the eyelid, often with pain & swelling; granulomatous tissue and lipid‐filled macrophages.
Capillary hemangioma	Red or bluish—red vascular lesions; Proliferation of capillary blood vessels.
Sebaceous adenoma	Raised, smooth, yellowish nodules on the skin; Adenomatous proliferation of sebaceous gland cells.
Juvenile Xanthogranuloma	Yellow‐orange papules or nodules, commonly in children; Proliferation of histiocytes with foamy cytoplasm and touton giant cells.
Rhabdomyosarcoma	Soft tissue mass with rapid growth, pain, or bleeding; malignant tumor showing evidence of skeletal muscle differentiation.

The final diagnosis of Pilomatricoma is based on the histopathological findings. Fine needle aspiration cytology (FNAC) can also be used as a preoperative diagnostic tool.^10^, ^11^ The radiological techniques are least helpful but ultrasonography, computed tomography (CT) and magnetic resonance imaging (MRI) can aid in preoperative diagnosis.^7^ Histopathological examination shows peripheral islands of basaloid cells and central areas of shadow cells.^8^ The basaloid cells are basophilic round cells with centrally located prominent vesicular nucleus and few cytoplasm.^12^ As the tumor matures, its constituent cells undergo changes. They acquire more cytoplasm, lose their nuclei, and transform into eosinophilic cells known as “shadow” or “ghost” cells. Additionally, in long‐standing cases of Pilomatricoma, areas of calcification and ossification may also become visible within the tumor.^6^

In 1931, Rywkind and Shiltzow initially termed ghost cells as “Rote Zellen,” a name originally applied to the undifferentiated cord of craniopharyngiomas. Later, Rywkind proposed a new name, “Verhornte epithelzellen,” which translates to keratinized epithelial cells, indicating the nature and origin of the ghost cells.^13^ It was first described in Pilomatricoma by Highman and Ogden as dyskeratotic cells in 1936.^13^, ^14^ In 1937, Robinson introduced the term “ghost cells” to describe the degenerative changes observed in the stellate reticulum cells during the formation of microcysts.^13^

Ghost cells are pale, eosinophilic having swollen cytoplasm and absence of nucleus. The term ghost cell was derived due to its hazy or shadowy, indistinct and structure less appearance in the H and E‐stained sections. The ghost cells are originated from the epithelium and these are found in the groups without intercellular junctions particularly in the thicker areas of the epithelial lining.^13^, ^14^, ^15^ The various theories^14^, ^15^, ^16^, ^17^ regarding the formation of ghost cells have been summarized in a Table 2. Ghost cells can be found in several conditions such as calcifying odontogenic cysts, Pilomatricoma, dentinogenic ghost cell tumors, ghost cell odontogenic carcinomas, odontomas, ameloblastomas, ameloblastic fibromas, and craniopharyngiomas.^14^, ^15^

**TABLE 2:** Different hypothesis for ghost cell formation.

Author(s)	Hypothesis
Hashimoto et al.	Ghost cell represents cells in advanced stages of keratinization
Praetorious	Ghost cells have abnormal keratinization and may undergo calcification.
Gorlin et al.	Ghost cells encompass both normal and aberrantly keratinized cells, representing squamous metaplasia with calcification possibly due to ischemia.
Sedano, Pindborg, Kerebel et al.	Ghost cells represent various stages of keratin formation resulting from metaplastic transformation due to loss of developmental and inductive influences.
Hong et al.	Ghost cells are formed due to coagulative necrosis and show weaker or unreactive staining compared to adjacent odontogenic epithelium with peroxidase‐antiperoxidase staining methods using polyclonal antikeratin antibody.
Kim et al.	Formation of ghost cells is due to the apoptotic process, evidenced by the expression of Bax protein and absence of Bcl‐2.

Histopathological analysis for Pilomatricoma often involves distinguishing it from several other skin conditions. These include basal cell carcinoma, tricholemmal cysts, trichoblastoma, and pilomatrix carcinoma (Table 3).^9^

**TABLE 3:** Histopathological differential diagnosis.

Condition	Histopathological Features
Basal cell carcinoma	Palisading arrangement of basaloid cells, peripheral palisading, retraction artifact, and clefting.
Tricholemmal cyst	Cyst lined by stratified squamous epithelium with abrupt keratinization, without shadow cells.
Trichoblastoma	Islands or nests of basaloid cells with metrical differentiation, without shadow cells.
Pilomatrix carcinoma	Invasive growth pattern, cytologic atypia, mitotic figures, and infiltrative margins.

Shimzu et al. conducted immunohistochemical studies and observed the expression of HKN‐2, HKN‐4, and HKN‐7 cytokeratin's within the epithelial aggregates. These findings mirror the cytokeratin expression pattern typically found in the inner layers of the cortex of the hair shaft, thereby confirming the metrical differentiation characteristic of Pilomatricoma.^12^ Reigner et al. and Cribier et al. identified the expression of keratin hHb1, which is typically found in intermediate cells.^18^, ^19^ This discovery provides additional evidence supporting the differentiation of cells within this neoplasm into cells resembling those of the hair cortex. The presence of Osteopontin and BMP‐2 expression in shadow cells or ghost cells provides further evidence supporting their role in the formation of areas of calcification and ossification within Pilomatricoma. Trichohyalin, along with its related proteins like Filaggrin and Involucrin, are detected in intermediate cells. It is believed that these proteins play a role in the transformation of these cells into keratinized shadow or ghost cells within pilomatricoma.^12^

Table 4 summarizes the different treatment options for pilomatricoma.^3^, ^6^ The primary treatment for Pilomatricoma involves wide surgical excision with 1–2 cm of clear margins to alleviate the risk of malignant transformation and recurrence at the site.^1^ Additional treatment modalities mentioned in Table 4 include punch incision and curettage.^16^ Erol et al. reported a similar case involving a 44‐year‐old female with a painless, mobile mass palpable under her right eye, which was managed with complete surgical excision. The patient underwent a 24‐month follow‐up period without experiencing any signs or symptoms of recurrence.^1^ In cases of giant Pilomatricoma, the surgical excision often results in a large defect that requires reconstruction. Local flaps are advantageous for this purpose due to their excellent color matching. Nadershah et al. reported a case involving a 28‐year‐old male with a Pilomatricoma presenting as a left facial mass. Following complete surgical excision, the resulting large cheek defect was successfully reconstructed using a cervicofacial flap.

**TABLE 4:** Treatment modalities.

Treatment	Description
Observation	Monitoring the lesion for changes over time.
Surgical excision	Complete removal of the tumor through surgery.
Mohs micrographic surgery	A precise surgical technique to remove the tumor layer by layer, minimizing damage to surrounding tissue.
Cryotherapy	Freezing the tumor with liquid nitrogen to destroy it.
Laser ablation	Using laser energy to destroy the tumor tissue.
Intralesional injections	Injection of medications directly into the tumor to shrink it.

The recurrence rate is very low and the incidence of recurrence is around 0%–3%.^1^, ^2^, ^11^ The malignant transformation of Pilomatricoma is very rare but malignant transformation can be suspected in cases showing recurrences.^1^, ^6^ The literature reports three documented cases of pilomatrix carcinoma arising in areas previously excised for pilomatricoma.^4^

Pilomatricoma presents a diagnostic challenge due to its rarity and varied clinical presentations. It is essential for clinicians to be well‐informed about this lesion. Careful screening and thorough clinical examination are crucial for identifying suspected cases, which should then be confirmed through histopathological examination for an accurate diagnosis. Early detection and treatment are essential for achieving better cosmetic outcomes and minimizing the risk of malignant transformation. Wide surgical excision is the preferred treatment to minimize the chances of local recurrence and prevent malignant transformation from the existing lesion.

## 6 PATIENT PERSPECTIVE

The patient will likely appreciate the treatment's effectiveness in removing the lesion and preventing recurrence or malignant transformation. They will also value the aesthetic outcomes of the reconstruction, particularly the use of local flaps for better color matching and minimal scarring.

## 7 CONCLUSION

Pilomatricoma presents a diagnostic challenge due to its rarity and variable clinical presentation. It is crucial for clinicians to be well‐informed about this lesion and maintain a high level of suspicion, especially when encountering subcutaneous nodules or masses in patients. Careful screening and thorough clinical examination are essential steps in identifying suspected cases, followed by confirmatory histopathological examination for accurate diagnosis.

Early detection is key to prompt and effective treatment. Complete surgical excision with clear margins is the mainstay of treatment for Pilomatricoma. Adequate surgical removal not only ensures accurate diagnosis but also leads to favorable outcomes and reduces the risk of recurrence.

## AUTHOR CONTRIBUTIONS

**Dilasha Dhungel:** Conceptualization; data curation; formal analysis; investigation; methodology; project administration; resources; writing – original draft; writing – review and editing. **Varun Rastogi:** Conceptualization; data curation; formal analysis; project administration; resources; supervision; validation; visualization; writing – original draft; writing – review and editing. **Sandhya Chaurasia:** Conceptualization; methodology; project administration; software; writing – original draft; writing – review and editing. **Nisha Maddheshiya:** Conceptualization; formal analysis; methodology; supervision; validation; writing – original draft; writing – review and editing.

## FUNDING INFORMATION

The authors did not receive support from any organization for the submitted work.

## CONFLICT OF INTEREST STATEMENT

The authors declare that they have no conflict of interest.

## ETHICAL APPROVAL

Our institution does not require ethical approval for reporting individual cases.

## CONSENT

Written informed consent was taken from the patient for their anonymized information to be published in this article.

## Data Availability

Data sharing not applicable to this article as no datasets were generated or analyzed during the current study.
